# Reemergence and Autochthonous Transmission of Dengue Virus, Eastern China, 2014

**DOI:** 10.3201/eid2109.150622

**Published:** 2015-09

**Authors:** Wen Wang, Bin Yu, Xian-Dan Lin, De-Guang Kong, Jian Wang, Jun-Hua Tian, Ming-Hui Li, Edward C. Holmes, Yong-Zhen Zhang

**Affiliations:** State Key Laboratory for Infectious Disease Prevention and Control, National Institute for Communicable Disease Control and Prevention, Chinese Center for Disease Control and Prevention, Beijing, China (W. Wang, M.H. Li, E.C. Holmes, Y.Z. Zhang);; Collaborative Innovation Center for Diagnosis and Treatment of Infectious Diseases, Hangzhou, China (W. Wang, M.H. Li, Y.Z. Zhang);; Wuhan Center for Disease Control and Prevention, Wuhan, China (B. Yu, D.G. Kong, J.H. Tian);; Wenzhou Center for Disease Control and Prevention, Wenzhou, China (X.D. Lin, J. Wang);; The University of Sydney, Sydney, New South Wales, Australia (E.C. Holmes).

**Keywords:** Dengue virus, viruses, phylogeny, evolution, autochthonous transmission, vector-borne infections, *Aedes aegypti*, mosquitoes, Fujian, Thailand, Surinam, India, Bangladesh, Sri Lanka, China

## Abstract

In 2014, 20 dengue cases were reported in the cities of Wenzhou (5 cases) and Wuhan (15 cases), China, where dengue has rarely been reported. Dengue virus 1 was detected in 4 patients. Although most of these cases were likely imported, epidemiologic analysis provided evidence for autochthonous transmission.

Four dengue viruses (DENV-1–4) circulate globally (*1*), each associated with either clinically mild dengue fever or, less frequently, with severe disease syndromes including hemorrhagic fever. Dengue is highly prevalent in tropical and subtropical regions, reflecting the distribution of the vector, *Aedes aegypti* mosquitoes. Nearly one third of the global human population is at risk for infection (*2*).

Dengue outbreaks were recorded in China during World War II (*3*). The disease then was not reported for ≈30 years, and reemerged during the late 1970s in Guangdong Province, located in the far south end of the country (*4*). Since then, dengue has been reported each year in China, mainly in Guangdong Province and its neighboring provinces (*4*,*5*). The geographic restriction of dengue to these southern provinces likely reflects temperature constraints in the range of *A. aegypti* mosquitoes. However, increasing travel has resulted in imported dengue cases in other provinces, including northern temperate regions (*6*,*7*), and some instances of autochthonous transmission (*8*).

During 2014, a dengue epidemic occurred in southern China (Figure 1); >40,000 cases were reported (*5*,*9*). This outbreak led to an increase in dengue surveillance in tropical and subtropical regions of China. We describe 20 dengue cases in the eastern coastal city of Wenzhou in Zhejiang Province and in Wuhan, the capital city of Hubei Province in the eastern central region of China (Figure 1), where the virus has rarely been described.

**Figure 1:**
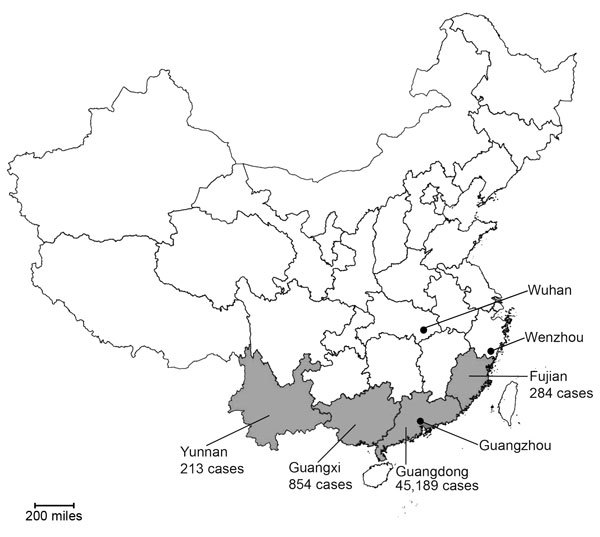
Geographic distribution of dengue cases reported during the 2014 epidemic in China, showing the location of the cities of Wenzhou, Zhejiang Province, and Wuhan, Hubei Province, in comparison to the focal area of the epidemic in southern China (Yunnan Province, Guangxi Zhuang Autonomous Region, Guangdong Province, and Fujian Province; gray shading). Case counts are shown for provinces in the focal area.

## The Study

During July–November 2014, a total of 20 suspected cases of illness were clinically diagnosed as dengue in Wenzhou (5 cases) and Wuhan (15 cases). The cohort comprised 14 male and 6 female patients 7–61 (median 31) years of age. Of the patients from Wuhan, 11 had recently traveled to Guangdong Province; 3 had recently returned from Indonesia and 1 from Thailand after >1 year away from China. Similarly, 3 of the patients from Wenzhou had traveled recently in Fujian, Thailand, and Surinam. However, there was no evidence of recent travel to endemic regions for the remaining 2 patients, including a 7-year-old boy. Close contacts of these patients denied recent travel to endemic regions, suggesting autochthonous dengue virus transmission in Wenzhou.

Blood samples from each of the 20 patients were collected on day 1 of hospitalization (2–4 days after onset of fever), and were tested for DENV IgM by a non–serotype-specific dengue dual IgM- and IgG-capture ELISA Kit (PanBio, Windsor, NSW, Australia). ELISA results showed 16 serum samples were positive for dengue-specific IgM and 3 for dengue-specific IgG. Although the remaining 4 serum samples were negative for dengue-specific IgM, we amplified DENV sequences from them, as described in the next section. Results of routine microbiologic examinations for bacteria by culture and antigen detection were negative in all cases, as were serologic and genetic tests for hantaviruses, phleboviruses, and *Rickettsiales* bacteria, performed as described (*10*).

The 20 dengue case-patients showed a variety of clinical symptoms (Table): high fever (100%), headache (100%), dizziness (45%), myalgia (50%), nausea and vomiting (40%), rash (40%), and petechiae (25%). In addition, chills, arthralgia, anorexia, enlarged lymph nodes, cough, and diarrhea were observed in some patients, and most displayed leucopenia (60%) and thrombocytopenia (65%). However, none showed plasma leakage, severe bleeding, or severe organ involvement. All patients recovered within a week of admission.

**Table:** Clinical characteristics of patients who had dengue fever, eastern China, 2014

Clinical feature	Positive PCR or antibody test result, n = 20	Location	
		Wenzhou, n = 5	Wuhan, n = 15
Fever	20	5	15
Headache	20	5	15
Dizziness	9	1	8
Chills	2	0	2
Myalgia	10	2	8
Arthralgia	2	1	1
Nausea and/or vomiting	8	2	6
Anorexia	4	0	4
Enlarged lymph nodes	3	0	3
Cough	4	0	4
Diarrhea	2	0	2
Rash	8	3	5
Petechiae	5	2	3

Total RNA was extracted from all blood samples as described by Chen et al. (*10*). Viral RNA in blood samples from individual patients was detected by reverse transcription PCR based on the conserved regions of the E gene (*11*). Consequently, dengue viral RNA was recovered in serum samples from 4 travel-associated patients with dengue (1 from Wenzhou and 3 from Wuhan) within 6 days after onset of disease, but not in the remaining serum samples. By using 24 pairs of primers, complete genome sequences were amplified successfully from the serum samples of 4 patients, all of which were characterized as DENV-1 (Technical Appendix Figure). The complete genome of all 4 viruses was 10,703 nt, and the isolates showed very high (99.9%) sequence identity to each other.

**Figure 2:**
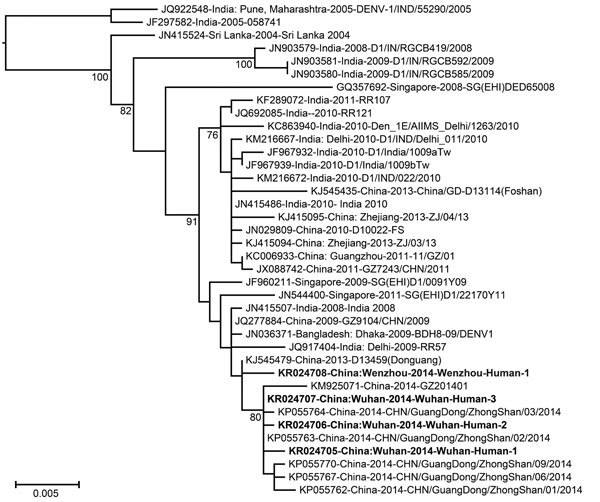
Phylogenetic analysis of a subset of dengue virus 1 E gene sequences within genotype III that are most closely related to those sampled from Wenzhou, Zhejiang Province, and Wuhan, Hubei Province, China, during 2014. The viruses identified in this study were designated as the Wenzhou-human and Wuhan-human sequences, respectively (GenBank accession nos. KR024705–KR024708). Bootstrap values (>70%) are shown at relevant nodes. Bold text indicates sequences obtained in this study. The tree is midpoint rooted for clarity. Scale bar indicates nucleotide substitutions per site.

Using the maximum-likelihood method implemented in PhyML v3.0 (*12*), we estimated phylogenetic trees based on the complete E gene or whole genome sequences of the 4 viruses identified in China and reference sequences from GenBank (Figure 2; Technical Appendix Figure). As expected, the viruses we identified are most closely related to those isolated in Guangdong Province in 2014, indicating they are part of the same outbreak, although with independent incursions into Wenzhou and Wuhan. The remainder of the phylogenetic trees show a mix of viruses from China and the Indian subcontinent (India, Bangladesh, Sri Lanka), indicative of the movement of viruses among these localities, as well as a small number from Singapore. However, because DENV sequences were only recovered from 4 patients, our molecular epidemiologic analysis was limited in scope, making extensive viral sampling necessary to reveal detailed transmission routes.

## Conclusions

Dengue has been relatively commonly reported in China, mainly in the southern provinces (*4*,*13*). Although the sustained transmission of DENV is possible in these localities, many cases appear to have resulted from importation from countries in Southeast Asia (*8*,*13*,*14*). In contrast, DENV has been sporadically reported in other regions of China, and those cases have been strongly associated with importation (*6*–*8*). Epidemiologic, serologic, and virologic investigations all confirmed the presence of dengue in Wenzhou and Wuhan, even though dengue has not been reported from either region for several decades. Although 90% of patients had a recent history of travel to dengue-endemic areas within and external to China, 2 patients from Wenzhou had no recent travel history to regions in which dengue was endemic, suggesting the occurrence of autochthonous transmission.

Although all 4 DENVs have been identified in China in recent years, DENV-1 appears to be the most common (*4*,*5*,*13*) and was observed in this study (Figure 2). These viruses were most closely related to those from Guangdong province, where the greatest number of cases were identified during the 2014 epidemic (Figure 1).

The viruses in this study were most closely related to those from the Indian subcontinent. Although India likely has the highest dengue incidence globally (*15*) and is therefore expected to harbor high levels of genetic diversity, the viruses endemic to India were identified >3 years before those found in China. Hence, although it is possible that the DENV-1 viruses in China originated in India and made multiple incursions in recent years, limited sampling in other localities, notably parts of Southeast Asia, mean that the exact origins of the viruses found in China remain uncertain. Finally, sequences recovered during this study from Wenzhou and Wuhan and from Guangdong Province in 2014 are very closely related to a virus isolated in Guangdong Province in 2013 that is likely to be related to the 2014 cluster. Although little is known about this latter virus, it will be critical to determine whether the 2014 epidemic directly arose from local ancestors present in 2013, rather than being imported.

This and previous studies (*6*,*8*) highlight the increasing risk that DENV-infected travelers may pose to public health in China. In humid subtropical regions such as Wenzhou and Wuhan, in which *A. albopictus* mosquitoes circulate with often poor control measures, imported dengue viruses may infect vector populations during permissive climatic conditions. Comprehensive strategies should be used to prevent the circulation of DENV among local *Aedes* mosquitoes.

**Technical Appendix.** Phylogenetic tree of whole genome sequences of dengue virus 1 isolated in the cities of Wenzhou, Zhejiang Province, and Wuhan, Hubei Province, China, 2014.
