# Supplementary material for: Reemergence and Autochthonous Transmission of Dengue Virus, Eastern China, 2014
Source: Emerg Infect Dis. 2015 Sep;21(9):1670–3. doi: 10.3201/eid2109.150622 (PMC4550164; doi:10.3201/eid2109.150622)
Supplement: Supplementary file 1 — Technical Appendix. Phylogenetic tree of whole genome sequences of dengue virus 1 isolated in the cities of Wenzhou, Zhejiang Province, and Wuhan, Hubei Province, China, 2014. [file 15-0622-Techapp-s1.pdf]

Article DOI: http://dx.doi.org/10.3201/eid2109.150622

Reemergence and Autochthonous Transmission of Dengue Virus, Eastern China, 2014

Technical Appendix

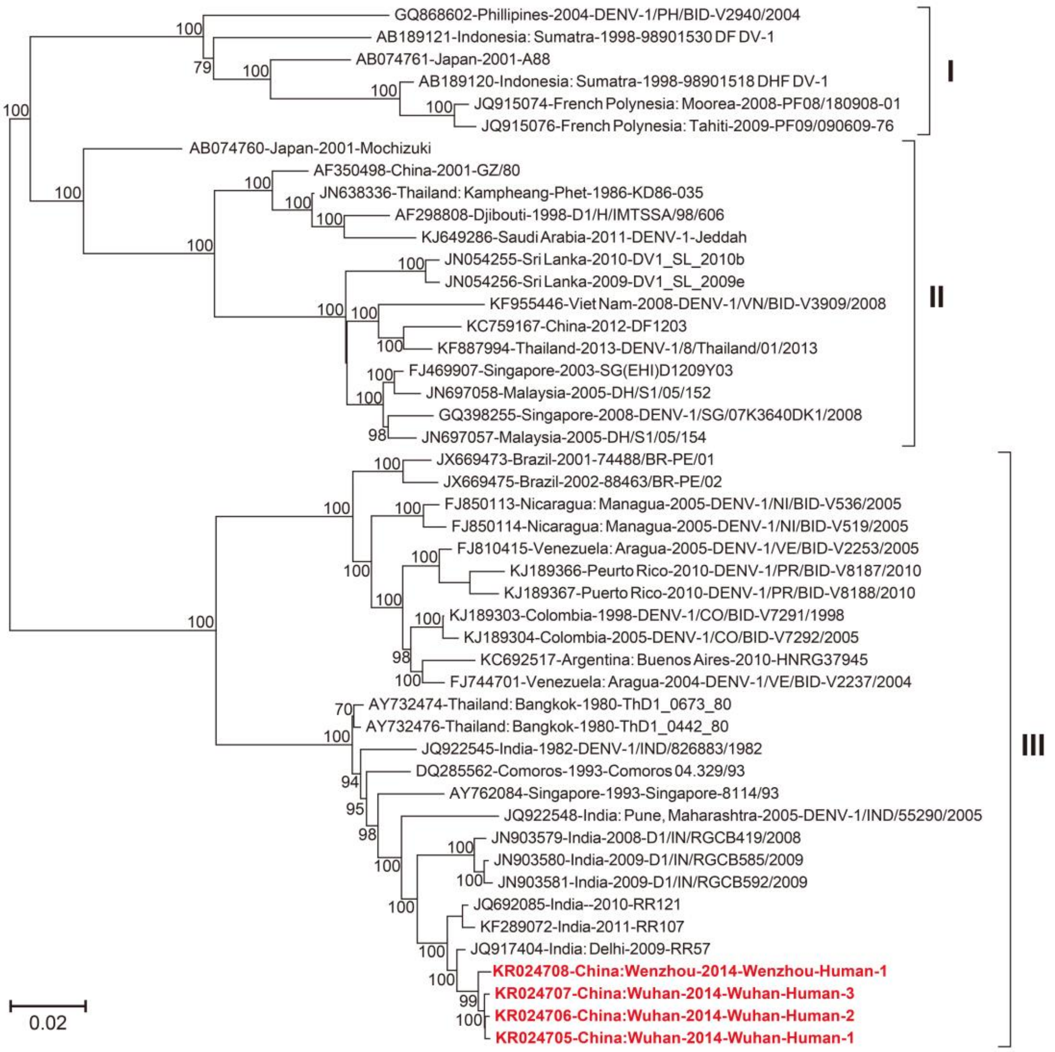

Technical Appendix Figure. Phylogenetic tree of whole genome sequences of DENV-1 showing the position of the Wenzhou and Wuhan viruses within viral genotype III. Bootstrap values (>70%) are shown at relevant nodes. The tree is mid-point rooted for clarity. Scale bar indicates nucleotide substitutions per site.
